# Are immediate postoperative X-Rays valuable in evaluating complications of primary total hip arthroplasty?

**DOI:** 10.1186/s42836-022-00148-1

**Published:** 2022-11-02

**Authors:** Matthew L. Brown, David Michel, Arvind Narayanan, Julie C. McCauley, William D. Bugbee

**Affiliations:** 1 Department of Orthopedic Surgery, Cooper University Health Care, Cooper University Hospital, 3 Cooper Plaza, Camden, NJ USA; 2 Austin Regional Clinic, Austin, TX USA; 3 Department of Orthopaedic Surgery, Scripps Green Hospital, La Jolla, San Diego, CA USA; 4 Shiley Center for Orthopaedic Research and Education at Scripps Clinic, La Jolla, San Diego, CA USA

**Keywords:** Total hip arthroplasty (THA), Complications, Radiographs, Value, Safety

## Abstract

**Purpose:**

This study aimed to investigate the complications of primary total hip arthroplasty based on immediate postoperative X-rays. The overall quality and cost of X-rays were assessed.

**Methods:**

The institutional database was queried to identify all patients who underwent total hip arthroplasty in a single institution between January 1, 2018, and December 31, 2018. Immediate postoperative X-rays were reviewed to identify the complications such as periprosthetic fractures, dislocation, and fixation failure. The quality and cost of X-ray were assessed. The complications were categorized as "known" and "unknown" according to the intraoperative fluoroscopic results.

**Results:**

A total of 518 total hip arthroplasties were included in this study. Based on intraoperative fluoroscopy, periprosthetic fractures were found in 10 (2%) THAs. Compared to the X-rays taken immediately after surgery, 9 periprosthetic fractures (recorded as "known") were found and 1 was not (recorded as "unknown"). There was no significant difference between intraoperative fluoroscopy and X-rays (*P* > 0.05). Of the 518 X-rays, 225 (43%) were of suboptimal quality. The cost of a single portable pelvic X-ray was $647.

**Conclusion:**

In total hip arthroplasty, X-rays taken immediately after surgery rarely reveal unknown complications. The X-rays are often of suboptimal quality, have minimal clinical utility, and are less cost-effective.

## Introduction

Periprosthetic fractures and dislocations following total hip arthroplasty (THA) are rare catastrophic complications [1–6]. Immediate postoperative X-rays are often performed in the post-anesthesia care unit (PACU) to screen for complications and assess implant placement, but their effectiveness and cost-effectiveness remain controversial.

Some surgeons often take immediate postoperative X-rays using a portable machine. Mulhall *et al*. [7] suggested that X-rays were not significantly superior to clinical assessments in diagnosing acute hip dislocations and that no dislocation occurred in clinical practice. Ndu *et al*. [8] advocated the use of criteria to determine whether X-rays were adequate. They found that 13% of anteroposterior X-rays and 6% of lateral X-rays were inadequate. Glaser *et al*. [9] argued that immediate postoperative X-rays were not cost-effective because they neither provide additional clinically relevant information nor benefit patient care.

This study aimed to investigate THA complications based on the immediate postoperative X-rays taken in the PACU. We also assessed the quality and cost of the X-rays associated with this protocol.

## Materials and methods

We retrospectively reviewed the data collected prospectively from our institutional arthroplasty database. The database was queried to identify all patients undergoing THA between January 1, 2018 and December 31, 2018. The inclusion criteria for the study were (1) patients undergoing THAs through the posterolateral or direct anterior approach; and (2) patients taking a standard anteroposterior pelvic X-ray using a portable radiograph machine in the PACU immediately after surgery. The exclusion criteria included simultaneous bilateral THAs, conversion THAs, THAs due to fractures, revision THAs, and a follow-up period of less than 3 months.

All operations were performed by 5 high-volume arthroplasty surgeons. One surgeon used both the direct anterior and posterolateral approaches, and four surgeons used the posterolateral approach. The attending surgeons selected the implants.

### Radiographic assessment

A fellowship-trained arthroplasty surgeon reviewed all PACU X-rays taken immediately after surgery. Complications were defined as hip fractures, dislocations, and fixation failure. We did not assess leg length, offset, component size, or implant position because the technical parameters were beyond the scope of this study. The complications were classified into "known" and "unknown" according to the intraoperative fluoroscopic results recorded in the operative notes.

### X-ray quality assessment

Suboptimal radiographic quality was confirmed based on one or more of the following: (1) malpositioned pelvic X-rays; (2) X-rays of incomplete pelvic, bilateral proximal femurs, and implants; (3) excessive abduction precluding an accurate assessment of leg length; (4) pelvic inlet, outlet, or Judet views due to significant malrotation; and (4) the presence of foreign body obscuring relevant anatomical structures. A random sample of 50 pelvic X-rays was independently reviewed by a second fellowship-trained arthroplasty surgeon.

### Cost assessment

The billing data for portable pelvic X-rays were assessed. As described by Longenecker *et al*. [10], the direct and associated costs for taking a single postoperative X-ray were estimated from the reimbursement data. As previously described by ourselves and other surgeons, intraoperative fluoroscopy was routinely performed in patients treated through the posterolateral approach [11–13].

### Statistical analysis

Patients’ data were recorded as mean and standard deviation. We compared the complication rates using Fisher’s exact Chi-square test. Statistical significance was set at *P* < 0.05. Inter-rater reliability was calculated. Analyses were performed using IBM SPSS Statistics for Windows version 28.0 (IBM Corp, Armonk, NY, USA).

### Source of funding

This study was supported by a Scripps Clinic Medical Group research grant.

## Results

**Fig. 1:**
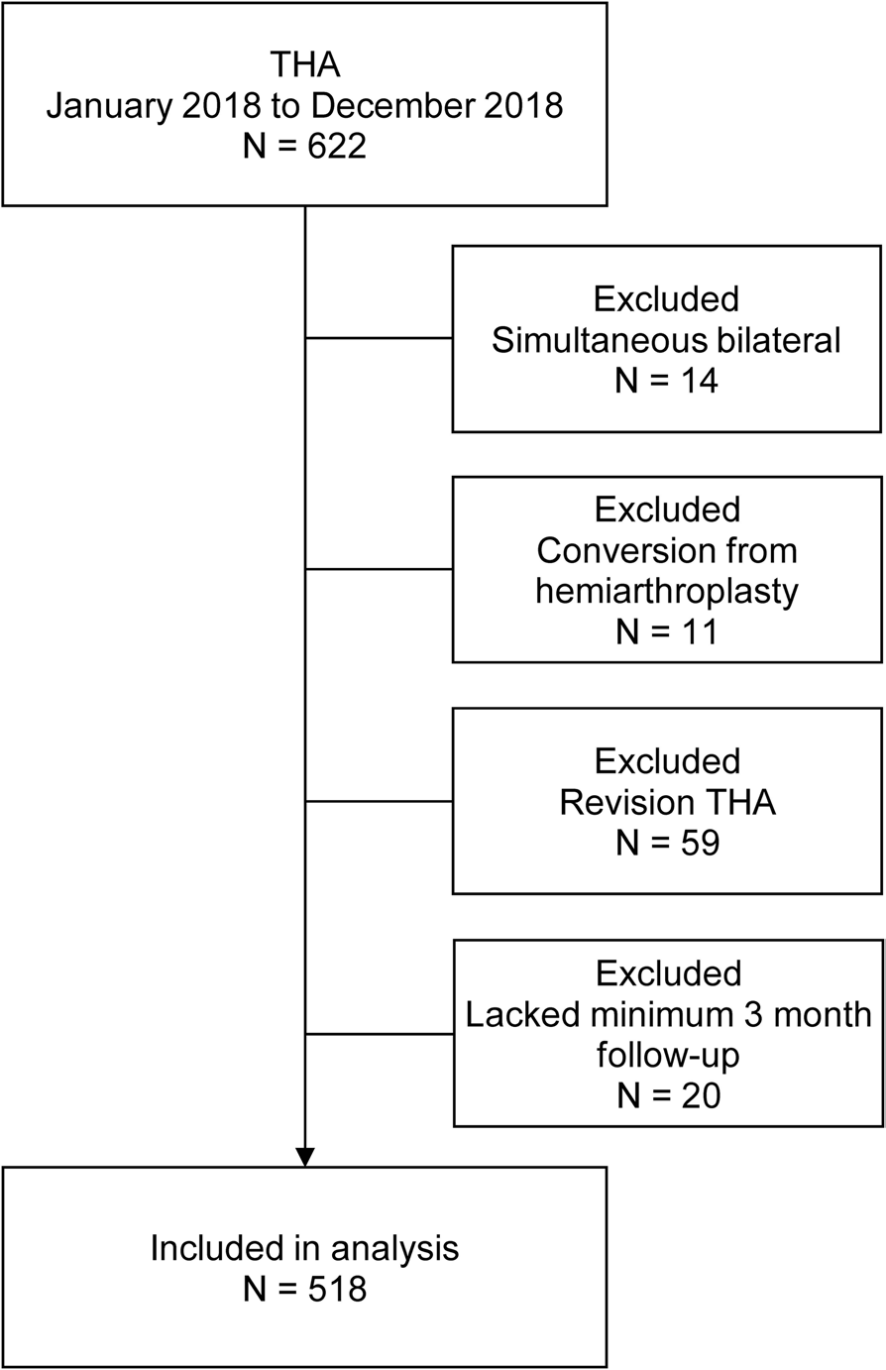
Patient flowchart

There were 563 THAs in the database, and 45 THAs were excluded. Thus, 518 THAs were included in this study (Fig. 1). The mean age at surgery was 67.2 years ± 12.1 years (range, 24 to 94 years). There were 288 female patients and 230 male patients. There were 514 (99%) cementless femoral stems and 4 (0.8%) cemented stems.

Based on the intraoperative fluoroscopic results, we found periprosthetic fractures occurred in 10 of 518 (2%) THAs. No hip dislocation was found. Compared to the X-rays taken immediately after surgery, 9 patients had complications (recorded as known) and 1 patient had not (recorded as unknown). In more detail, we used the direct anterior approach in 66 (13%) THAs, and 3 (5%) THAs had complications (3 known; 1 unknown); and we used the direct anterior approach in 452 (87%) THAs, and complications occurred in 7 (2%) THAs (7 known; 0 unknown). There was no significant difference between intraoperative fluoroscopy and X-rays taken immediately after surgery (*P* = 0.123).

Of the 518 PACU X-rays, 225 (43%) were of suboptimal quality. The flaws included excessive abduction (*n* = 70, 14%), malposition (*n* = 67, 13%), malrotation (*n* = 47, 9%), the presence of foreign bodies (*n* = 2, 0.3%), and multiple errors (*n* = 45, 9%). Owing to the suboptimal quality, the radiology technician decided to take a repeated X-ray in 21 (4%) patients. However, 10 of the 21 (48%) repeated X-rays were again rated as suboptimal quality. Inter-rater reliability was assessed with a kappa coefficient of 0.23, indicating comparable inter-observer agreement.

The cost of a single portable pelvic X-ray at our institution was $647. In 2021, the reimbursement for 1 to 2 X-ray(s) (current procedural terminology code 72,170) listed in the Center for Medicare and Medicaid Services fee schedule was $56.7. So, the total cost of 518 X-rays was estimated at $29,370.

## Discussion

In THA, we found that immediate postoperative X-rays taken in the PACU rarely showed unknown complications, and the quality of X-rays was often suboptimal. Therefore, PACU X-rays offer minimal clinical utility and are less cost-effective.

Immediate postoperative X-rays are often used to screen for unrecognized intraoperative complications, assess implant placement, and establish a radiographic baseline for follow-up. However, the value of PACU X-rays has been challenged, given the low incidence of serious complications and the limited quality in knee, hip, and shoulder arthroplasties [8–10, 14–18]. Furthermore, the cost-effectiveness is particularly questioned, although radiographic costs vary widely across the countries [9, 19]. Mulhall *et al*. [7] reviewed 2,065 THAs performed at a single institution over 10 years. They suggested the value of immediate postoperative X-rays because 6 (0.3%) dislocations were diagnosed in the recovery room. Among them, 4 dislocations were diagnosed clinically and radiographically, and 2 were clinically unsuspected and newly diagnosed on X-ray. They also compared the quality of PACU X-rays to the X-rays taken at 3-month follow-up visits, using a novel grading system that categorizes the quality as unreadable (7% *vs.* 0%), poor (68% *vs.* 1%), satisfactory (22% *vs.* 13%), and good (3% *vs.* 86%). Ndu* et al*. [8] assessed the complications and quality of X-rays on anteroposterior and lateral views. The X-rays were taken using a portable machine in the PACU immediately after surgery. In a series of 633 primary THAs (587 cementless THAs), they identified 12 (2%) complications, including dislocation (*n* = 1), medial screw penetration of the acetabular component (*n* = 7), acetabular protrusion (*n* = 1), and periprosthetic femur fractures (*n* = 3). Among them, only 2 complications were treated surgically (the dislocation was reduced, and the protruding crew was removed).

Our results corroborate Mulhall's findings but with different complications. We used larger femoral heads to prevent hip dislocation [3, 20], but our cementless fixation was associated with a higher incidence (5%) of acute periprosthetic fractures, compared to the incidences (0.1% to 1.0%) of cementless femoral fixation [1, 2, 21]. Many surgeons reported that THAs performed through the direct anterior approach had a significant learning curve and a higher risk of periprosthetic fractures, especially femoral fractures [22–25]. In our study, however, this trend was not statistically significant because we used this approach only in a few THAs. Moreover, the study was not specifically designed to compare the two approaches.

In PACUs, low-level portable X-ray machines may produce suboptimal X-rays. Other factors include difficulties in patient positioning secondary to pain, anesthetics, dressings, and braces. In addition, portable X-ray machines generate more radiation to many patients and medical staff around. The dose of a pelvic X-ray was approximately 0.7 mSv, which is 35 times the dose of a chest X-ray and is equivalent to 4-months of exposure to natural background radiation [26]. Nevertheless, PACU X-rays may benefit the surgeons who do not obtain intraoperative X-rays and low-volume surgeons who perform THA in low-volume hospitals.

This study has limitations that may affect the generalizability of its findings. First, we routinely obtain flat panel X-rays intraoperatively to assess trial components when performing THAs through the posterolateral approach. This practice improves our ability to diagnose the complications intraoperatively and reduces the "unknown" complications diagnosed on PACU X-rays. Second, we use intraoperative fluoroscopy when performing THAs through the direct anterior approach, which increases our ability to diagnose the complications. However, compared to flat panel X-rays, intraoperative fluoroscopy limits the detectability of fine and subtle features. Third, all THAs were performed by high-volume arthroplasty surgeons at a single high-volume hospital, which may affect the ability to identify the complications intraoperatively and the overall complication rate.

## Conclusion

In THA, immediate postoperative X-rays taken in the PACU rarely reveal unknown complications. The X-rays are often of suboptimal quality, have minimal clinical utility, and are less cost-effective. 

## Data Availability

The datasets during and/or analyzed during the current study are available from the corresponding author on reasonable request.

## Abbreviations

THA: Total hip arthroplasty
PACU: Post anesthesia care unit
